# Homogeneous Embedding of Magnetic Nanoparticles into Polymer Brushes during Simultaneous Surface-Initiated Polymerization

**DOI:** 10.3390/nano9030456

**Published:** 2019-03-19

**Authors:** Weronika Górka, Tomasz Kuciel, Paula Nalepa, Dorota Lachowicz, Szczepan Zapotoczny, Michał Szuwarzyński

**Affiliations:** 1 Faculty of Physics, Jagiellonian University, Astronomy and Applied Computer Science, S. Łojasiewicza 11, 30-348 Krakow, Poland; weronika.gorka@doctoral.uj.edu.pl; 2 Faculty of Chemistry, Jagiellonian University, Gronostajowa 2, 30-387 Krakow, Poland; kuciel@chemia.uj.edu.pl (T.K.); paula.nalepa@student.uj.edu.pl (P.N.); 3 Academic Centre for Materials and Nanotechnology, AGH University of Science and Technology, A. Mickiewicza 30, 30-059 Krakow, Poland; dbielska@agh.edu.pl

**Keywords:** polymer brushes, nanoparticles, SPION, thin magnetic films, ATRP, hybrid polymer/inorganic composites

## Abstract

Here we present a facile and efficient method of controlled embedding of inorganic nanoparticles into an ultra-thin (<15 nm) and flat (~1.0 nm) polymeric coating that prevents unwanted aggregation. Hybrid polymer brushes-based films were obtained by simultaneous incorporation of superparamagnetic iron oxide nanoparticles (SPIONs) with diameters of 8–10 nm into a polycationic macromolecular matrix during the surface initiated atom transfer radical polymerization (SI-ATRP) reaction in an ultrasonic reactor. The proposed structures characterized with homogeneous distribution of separated nanoparticles that maintain nanometric thickness and strong magnetic properties are a good alternative for commonly used layers of crosslinked nanoparticles aggregates or bulk structures. Obtained coatings were characterized using atomic force microscopy (AFM) working in the magnetic mode, secondary ion mass spectrometry (SIMS), and X-ray photoelectron spectroscopy (XPS).

## 1. Introduction

During the last decade great attention has been devoted to hybrid inorganic–polymer systems grafted or adsorbed to/from the surface [1,2] and their potential applications in cell culturing [3], fabrication of antibacterial coatings [4], sensors [5], photovoltaic devices [6], field-effect transistors [7], light-emitting diodes [8] and magnetic inks [9]. The incorporation of nanoparticles (NPs) offers advantages of combining properties of both entities: unique magnetic, electronic, thermal or optical properties of nanosized inorganic objects with physical and chemical properties of polymeric matrices [10,11,12]. A number of techniques could be used for controlled fabrication of well-defined hybrid nanocomposites such as: coating of inorganic particles by polymeric chains using “grafting to” and “grafting from” methods [13,14], layer-by-layer assemblies [4], in situ synthesis of NPs in between the surface-grafted polymer chains [15,16], the self-organization process of copolymer thin films blended with nanoparticles [17], and the polymerization of monomers in the presence of inorganic particles [18]. Polymer brushes [19], as coatings composed of chains tethered by one end to a surface, may be particularly useful matrices that work as a macromolecular capping agent for preventing aggregation due to the inherently extended chains’ conformation in the brushes. Because of that, polymer brushes, by linking a specific molecules or functional groups in demanded order, can be used for an advanced applications: conjugated side chains for conducive electricity [20,21], crown ethers for selective ion sensing [22], growth factors for cell culturing [23], or chromophore molecules for light absorption [24,25]. For better control on the size and composition of NPs it is often desired to form them ex situ and the polymer matrix volume should be limited for the formation of functional nanocomposites. There have been numerous attempts at incorporation of NPs into polymeric matrices but obtaining a thin layer with a thickness comparable to the diameter of the nanoparticles while maintaining their homogenous distribution in the layer is still a challenge [26,27,28] and typically much thicker polymer matrices have been used for that purpose [29,30]. 

Here we present an approach based on surface-initiated polymerization of a cationic monomer in the presence of superparamagnetic iron oxide nanoparticles (SPIONs) that leads to the formation of a magnetic nanocomposites layer with NPs homogenously distributed within the thin polyelectrolyte brushes. Such smooth magnetic ultrathin layers have high application potential but can hardly be obtained by embedding nanoparticles in already prepared brushes.

## 2. Materials and Methods

### 2.1. Materials

Superparamagnetic iron oxide nanoparticles (SPIONs): iron(III) chloride hexahydrate (p.a.), iron(II) chloride tetrahydrate (p.a.) were purchased from Sigma Aldrich (St. Louis, MO, USA). Ammonium hydroxide (25%, p.a.) was purchased from Chempur (Piekary Slaskie, Poland). 

Polymer brushes: (3-Aminopropyl)triethoxysilane (APTES, 99%), α-bromoisoubtyryl bromide (BIB, 98%), triethylamine (Et_3_N, ≥99.5%), N,N,N′,N′′,N′′-Pentamethyldiethylenetriamine (PMDETA, 99%), (3-acrylamidoproplyl)trimethylamonnium chloride (APTAC, 75% solution in water), copper (I) bromide (99.999%) were all purchased from Sigma Aldrich (St. Louis, MO, USA). Toluene (p.a.), methanol (p.a.), tetrahydrofuran (p.a,), isopropanol (p.a.), dichloromethane (high-performance liquid chromatography, HPLC grade, 99.8%) were purchased from Chempur (Piekary Slaskie, Poland). Polished Prime Silicon Wafers were obtained from Cemat Silicon SA (Warszawa, Poland) and ITO glass with the ITO layer thickness of 150 nm was purchased from VisionTek System LTD (Cheshire, United Kingdom). Ammonia solution (25% p.a.) and sulfuric acid (≥95% p.a.) were obtained from POCH S. A. (Gliwice, Poland). Hydrogen peroxide (30% p.a.) was purchased from Stanlab (Lublin, Poland).

### 2.2. Methods

A transmission electron microscope (TEM) Tecnai TF 20 X-TWIN (FEI, Hillsboro, OR, USA) was used for determination of the shape and the size of the nanoparticles. The aqueous dispersions of SPIONs were sonicated for 5 min before deposition on ultrathin carbon coated copper grid and air-dried at room temperature. The TEM images were analyzed by fitting round circles around the edges of the nanoparticles and measuring their diameters that lead to determination of the mean particle size. 

Sizes and zeta potentials of the nanoparticles were determined using dynamic light scattering (DLS, Malvern Nano ZS light-scattering apparatus, Malvern Instrument Ltd., Worcestershire, UK; measurements at 173° scattering angle) at 25 °C. The time-dependent autocorrelation function of the photocurrent was acquired every 10 s, with 15 acquisitions for each run. The sample was illuminated by a 633 nm laser. The z-averaged mean diameters (d_z_) and distribution profiles of the samples were collected using the software provided by Malvern. The zeta potential was measured using the technique of laser Doppler velocimetry (LDV). 

Atomic force microscope (AFM) images were obtained with a Dimension Icon atomic force microscope (Bruker, Santa Barbara, CA, USA) working in the air or water in the PeakForce Tapping (PFT) mode using standard silicon cantilevers of nominal spring constant of 0.4 N/m for air measurements and 0.7 N/m for liquids. Magnetic force microscope (MFM) images were acquired using the same microscope and magnetic Co/Cr coated standard silicon cantilevers of nominal spring constant of 2 N/m. All MFM images were captured in the lift mode at 50 nm lift height. A potential (5 V) between the tip and the sample was applied for the measurements of the brushes in order to compensate for their positive charge that could otherwise contribute to the magnetic phase signal. The cantilevers were magnetized with a small magnet before the measurements. Quantitative nanomechanical mapping (QNM) measurements were done using the previously calibrated silicon AFM tip. The results obtained were averaged for at least 10 locations on each sample. The thickness measurements were performed at the edges of the scratched layers.

Secondary ion mass spectrometry (SIMS) experiments were performed on an ION TOF TOFSIMS V (Munster, Germany) instrument, equipped with bismuth manganium liquid metal ion source and C_60_ ion source. The depth profiles of samples were obtained in interlaced dual beam mode; 20 keV C_60_^+^ ion beam was used to sputter a 500 × 500 μm^2^ area and Bi_3_^+^ 30 keV ion beam was used to analyse a 300 × 300 μm^2^ area concentric to the sputtered surface. For the surface characterization Bi_3_^+^ 30 keV ion beam was used with the ion dose density lower than 10^12^ ions/cm^2^ to ensure static SIMS conditions. TOF-SIMS spectra were acquired from 5 non-overlapping 500 × 500 μm^2^ area.

X-ray photoelectron spectroscopy (XPS) analysis were carried out in a PHI VersaProbeII Scanning XPS system (ULVAC-PHI, Chigasaki, Japan) using monochromatic Al Kα (1486.6 eV) X-rays focused on a 100 µm spot and scanned over the sample area of 400 × 400 µm. The photoelectron take-off angle was 30° and the pass energy in the analyser was set to 23.50 eV to obtain high energy resolution spectra for the C 1s, N 1s, O 1s, Cl 2p, Si 2p and Fe 2p regions. All XPS spectra were charged to the unfunctionalized, saturated carbon (C–C) C 1s peak at 284.8 eV. The pressure in the analytical chamber was less than 4 × 10^−8^ mbar. The deconvolution of spectra was performed using PHI MultiPak software (v.9.7.0.1). The spectrum background was subtracted using the Shirley method.

### 2.3. Synthesis of Superparamagnetic Iron Oxide Nanoparticles (SPIONs)

**Scheme 1:**
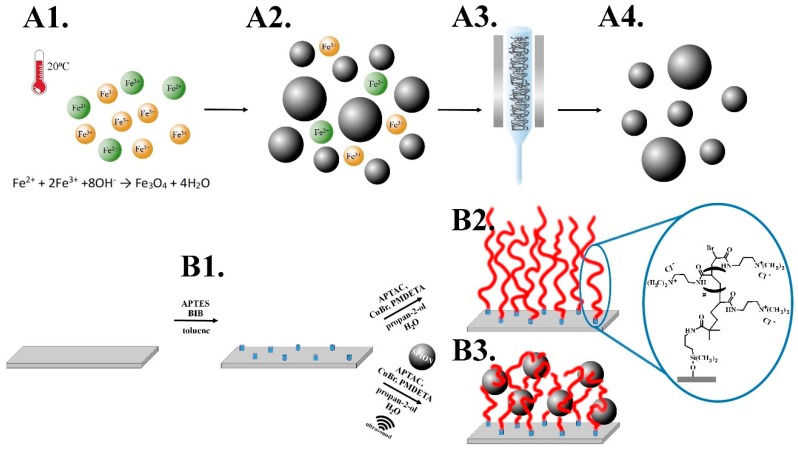
A. Synthesis of magnetic nanoparticles: 1. Fe^2+^, Fe^3+^ salts mixture at room temperature (RT) in H_2_O, 2. Mixture of nanoparticles and post-reaction remains, 3. Magnetic chromatography, 4. Purified nanoparticles. B. Synthesis of poly(APTAC) and poly(APTAC)+SPIONs brushes: 1. Amide-silane initiator adsorption on a silicon substrate, 2. Formation of poly(APTAC) brushes via surface initiated atom transfer radical polymerization (SI-ATRP) of APTAC, 3. Formation of poly(APTAC)+SPIONs via SI-ATRP of APTAC in the presence of the magnetic nanoparticles.

Briefly, the precursor solutions of the salts: 0.1622 g FeCl_3_·6H_2_O and 0.0596 g FeCl_2_·4H_2_O (the molar ratios of ions Fe(III): Fe(II) = 2:1, pH = 2.37) were dissolved in 50 mL of deionized water (Scheme 1A1). The solution was deoxygenated by purging with argon and sonicated (Sonic-6, Polsonic, Warszawa, Poland, 480 W, 1 s pulse per every 5 s) for 10 min in a thermostatic bath at 20°C. Afterward, 5 mL of 5 M NH_3(aq)_ were added dropwise, and the suspension of the formed polydisperse nanoparticles was further sonicated for 30 min (Scheme 1A2). Purification of the formed SPIONs was performed using magnetic chromatography (Scheme 1A3) until a stable dispersion of the nanoparticles without reaction remains was obtained (Scheme 1A4). 

### 2.4. Synthesis of Poly(APTAC) Brushes

Silicon wafers were firstly purified by immersing in a “piranha” solution (a mixture of H_2_SO_4_ and H_2_O_2_ at a 3:1 ratio) (Caution! “Piranha solution” should be handled with extreme care) for 1 h, subsequently rinsed by water and dried out in a stream of argon. Such prepared substrates were immediately immersed into the solution of amide-silane initiator (APTES, one drop in 5 mL of toluene) and left for 24 h in a sealed flask under argon atmosphere at room temperature. Afterwards the substrates were rinsed with copious amounts of toluene and dichloromethane. Then 2-isobromobutyryl bromide (BIB; 0.06 mL), triethylamine (Et_3_N; 0.07 mL) and dichloromethane (CH_2_Cl_2_; 10 mL) were added over the substrates under an argon atmosphere, at room temperature, and left for 1 h in a sealed flask (Scheme 1B1.). Poly(APTAC) brushes were obtained using the ATRP method. (3-acrylamidoproplyl)trimethylamonnium chloride monomer (APTAC; 3 mL, 75% solution in water) was added into the mixture of deionized water (0.375 mL) and isopropanol (1.125 mL) with dissolved CuBr (20 mg) under an argon atmosphere at room temperature. Then N,N,N′,N′′,N′′-pentamethyldiethylenetriamine (PMDETA; 0.1 mL) was injected into the deoxygenated solution, mixed and left for 72 h (Scheme 1B2.). The samples were then carefully cleaned in a mixture of water:propan-2-ol (1:1, *v*/*v*), toluene and methanol by bubbling for circa 5 min in each solution and finally dried in a stream of argon. 

### 2.5. Synthesis of Poly(APTAC) Brushes with Embedded SPIONs (Poly(APTAC)+SPIONs)

Brushes with incorporated magnetic nanoparticles were obtained similarly to the method described above but 0.2 mL of a stable dispersion of SPIONs in water was injected into a sealed reaction flask at the beginning of the ATPR reaction (Scheme 1B3.) and the first 1 h of the polymerization was conducted in the ultrasonic reactor (pulsed sonication as described in Section 2.3). Before injection, a solution of SPIONs was sonicated for 10 min (continuous sonication).

## 3. Results and Discussion

In the presented approach we first synthesized superparamagnetic iron oxide nanoparticles (SPIONs) with well-defined diameters of 8–10 nm (Figure S1, Supplementary Materials) and strong magnetic properties [31]. As shown in Scheme 1 the synthesis of SPIONs was carried out by coprecipitation of respective iron salts in an aqueous medium according to the method described previously [32,33]. The surface of the obtained SPIONs was shown to be strongly negatively charged (zeta potential, ξ = −47.6 ± 0.4 mV) so their suspension can be considered as stable (Figure S2, Supplementary Materials) that is crucial for the successful running of the polymerization. Moreover, magnetic properties of the formed NPs were studied previously and confirmed here using magnetic force microscopy (MFM, Figure S3, Supplementary Materials). The concentration of iron in the suspension of SPIONs was calculated to be 0.87 mg/mL (Figure S4, Supplementary Materials).

**Figure 1:**
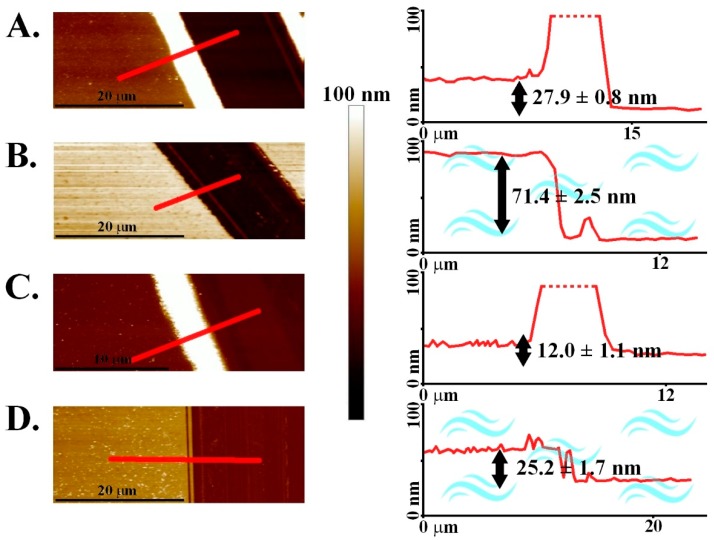
Atomic force microscopy (AFM) topography images of the brushes with the corresponding cross-sections: (**A**) (poly(APTAC) in the air, (**B**) poly(APTAC) in the water, (**C**) poly(APTAC)+SPIONs in the air, (**D**) poly(APTAC)+SPIONs in the water.

Polymer brushes were grafted from a silicone surface via surface initiated atom transfer radical polymerization (SI-ATRP) using (3-acrylamidoproplyl)trimethylamonium chloride (APTAC) as a monomer (see Scheme 1 and Materials and Methods for details). The brushes with incorporated SPIONs were obtained in a similar polymerization procedure but a stable dispersion of SPIONs was injected into a sealed reaction flask at the beginning of ATRP and for the first 1 h the process was conducted in an ultrasonic reactor (pulsed sonication). The obtained polycationic brushes (poly(APTAC)) and the same brushes with incorporated magnetic nanoparticles (poly(APTAC)+SPIONs), were characterized using AFM. Thicknesses of the dry coatings were determined to be 27.9 ± 0.8 nm and 12.0 ± 1.1 nm, respectively (Figure 1). 

**Figure 2:**
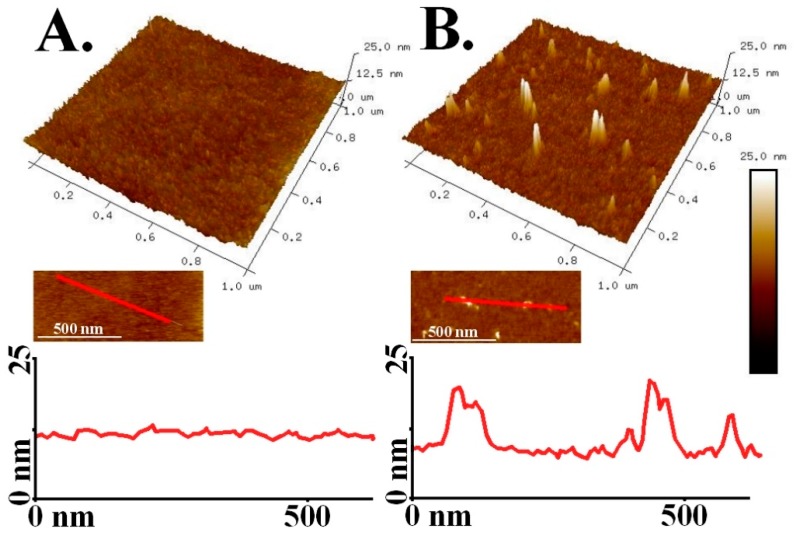
AFM images of poly(APTAC) brushes (**A**) and poly(APTAC)+SPIONs brushes (**B**) with the corresponding cross-sections.

The topography images (Figure 2) showed distinct differences between both layers. However, the calculated RMS roughness for poly(APTAC) brushes (0.8 ± 0.1 nm) was only slightly smaller than the value for the layer with incorporated SPIONs (1.2 ± 0.2 nm). There are a few objects sticking out from the smooth underlying layer in the poly(APTAC)+SPIONs sample. The AFM imaging indicates that while some nanoparticles sit on the top of the layer (features up to 10 nm above the layer), some other are partially or completely immersed in the brushes matrix. Importantly, practically no aggregates of SPIONs could be observed on the surface of poly(APTAC)+SPIONs thanks to the applied procedure. Moreover, the incorporation of SPIONs in the already prepared poly(APTAC) brushes, by depositing them under sonication, failed as indicated by the AFM measurements (Figure S5, Supplementary Materials). Stability over time of the obtained structures has been confirmed (see Figure S6, Supplementary Materials). After one month of storage at room temperature, no peeling process of brushes layer and no removal of SPIONs from the polymeric matrix was observed. 

**Figure 3:**
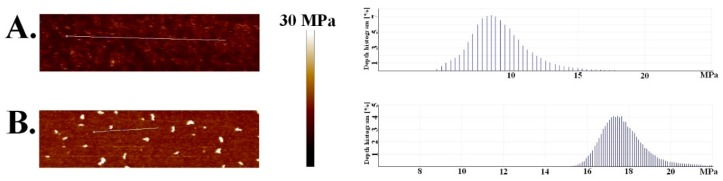
Quantitative nanomechanical mapping (QNM) measurements of poly(APTAC) (**A**) and poly(APTAC)+SPIONs (**B**) with the corresponding bearing analysis histograms.

**Figure 4:**
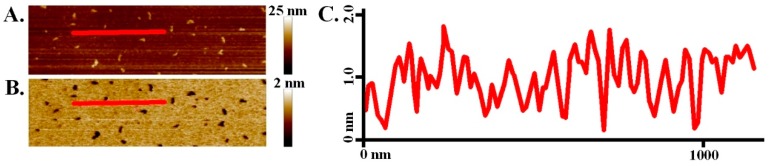
Poly(APTAC)+SPIONs brushes AFM images: topography (**A**) and indentation (**B**) with corresponding indentation image cross-section (**C**).

The results of the quantitative nanomechanical mapping (QNM) measurements may indicate efficient incorporation of the nanoparticles into the brush structure. The average DMT modulus (calculated using Derjaguin-Muller-Toporov model) [34] of poly(APTAC) brushes grafted from silicon was determined to be 8.8 ± 1.1 MPa, while for poly(APTAC)+SPIONs it was found to be twice as large (17.6 ± 1.3 MPa) (Figure 3). The reported values can be reliably compared as the average indentations applied during the QNM measurements were kept very small (ca. 1 nm for poly(APTAC)+SPIONs, Figure 4) limiting the influence of the underlying substrate. It seems that the incorporated nanoparticles that are wrapped by the grafted polymer chains significantly increase the modulus of the whole layer. There are clearly some spots on the surface of poly(APTAC)+SPIONs with much higher DMT modulus (116–173 MPa) that can be correlated with SPIONs located at the top of the brushes (see Figure 2B). The measured values are three orders of magnitude smaller than the values reported for SPION in literature (151–192 GPa) [35] indicating that they are supported here by soft brushes rather than adsorbed directly on the silicon surface. However, the AFM cantilever selected for the measurements of the soft polymer layer cannot be reliably used for the determination of elasticity of much harder inorganic nanoparticles so the values of DMT modules determined for the SPION sticking out from the surface should be treated with caution. It seems that the incorporated nanoparticles mechanically integrate the whole layer by cross-linking of the polymeric chains formed in their proximity to electrostatic interactions. 

**Figure 5:**
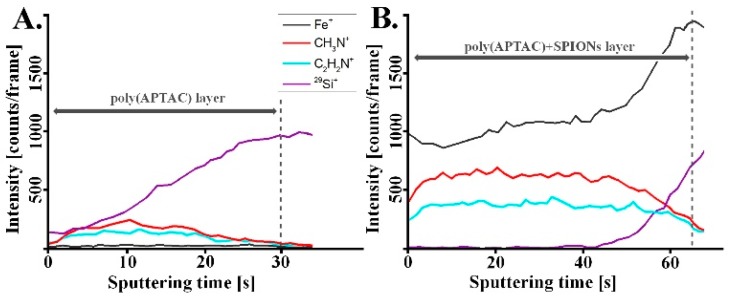
Secondary ion mass spectrometry (SIMS) spectra for polymeric brushes: (**A**) bare poly(APTAC), (**B**) poly(APTAC)+SPIONs.

The coating profiling using the secondary ion mass spectrometry (SIMS) confirmed distribution of SPIONs in the whole volume of the poly(APTAC)+SPIONs layer (Figure 5). The signals of CH_3_N^+^ and C_2_H_2_N^+^ ions, characteristic of poly(APTAC) brushes, appeared for the measurements of both coatings. Their intensities stay at the beginning constant during sputtering until they decrease when the signal of Si^+^ from the silicon substrate becomes more intense. For the poly(APTAC)+SPIONs the signal of iron from magnetic nanoparticles is clearly visible for the whole thickness profile while no such signal can be observed for poly(APTAC) brushes. Moreover, the Fe^+^ profile indicates homogeneous distribution of SPIONs along the brush thickness and even somehow higher content of SPIONs in the proximity of the silicon substrate. This observation is opposite to those of the systems prepared by incubation of polymer brushes in the suspension of nanoparticles for which the distribution is typically not homogeneous and even decreases gradually toward the substrate [28,36].

**Figure 6:**
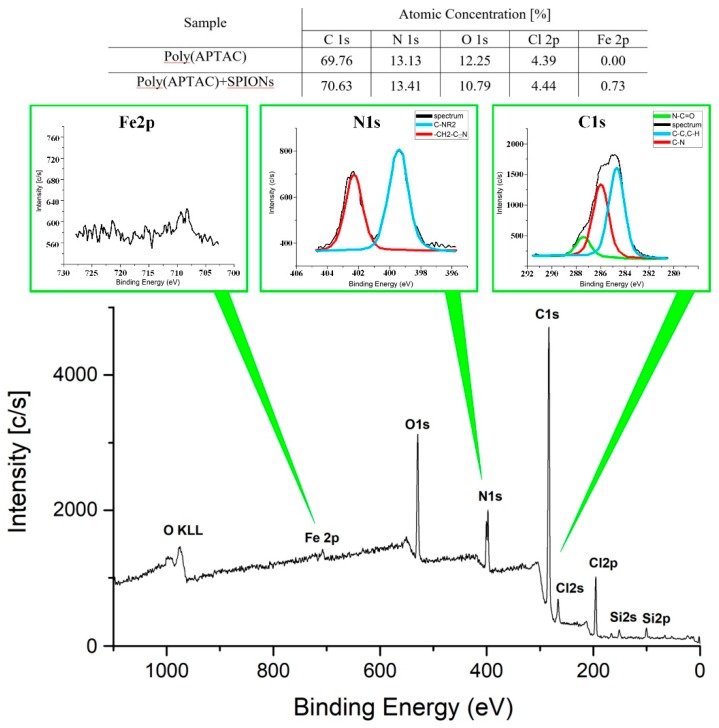
X-ray photoelectron spectroscopy (XPS) spectrum of poly(APTAC)+SPIONs with fragments corresponding to binding energies for Fe2p, N1s and C1s.

The amount of SPIONs loaded into the polymeric brushes was indicated using X-ray photoelectron spectroscopy (XPS). As shown in Figure 6 and Figure S7 (Supporting Materials) the bands with characteristic binding energies—C 1s: 284–286 eV, N 1s: 399–402 eV, O 1s: 531–533 eV—appeared for both samples. Moreover two bands—Cl 2s: 270 eV, Cl 2p: 200 eV—of chlorine that are present as counterions for positively charged poly(APTAC) are shown as well. The intensity of the band at 709 eV (Fe 2p^3/2^, iron from SPIONs) was used to estimate the total amount of iron in the sample to be ca. 0.7% (ca. 1% SPIONs) that correlates with a thin layer of SPIONs in the brushes. 

The thickness of the layers obtained was measured in both air and water using AFM (Figure 1). The polycationic chains are well-solvated in water that leads to their stretching due to repulsive interaction of the cationic side groups. This implies the appearance of poly(APTAC) brushes as a much thicker layer in water than in a dry state. In fact, the thickness of poly(APTAC) brushes increased more than 2.5 times reaching 71.4 ± 2.5 nm in water while for poly(APTAC)+SPIONs the increase was smaller reaching 2.1 times (thickness in water: 25.2 ± 1.7 nm). It seems that the embedded SPIONs limited the conformational freedom of the neighboring poly(APTAC) chains in the brushes due to mutual interactions and/or the formation of more entanglements of the chains that is consistent with the QNM results.

**Figure 7:**
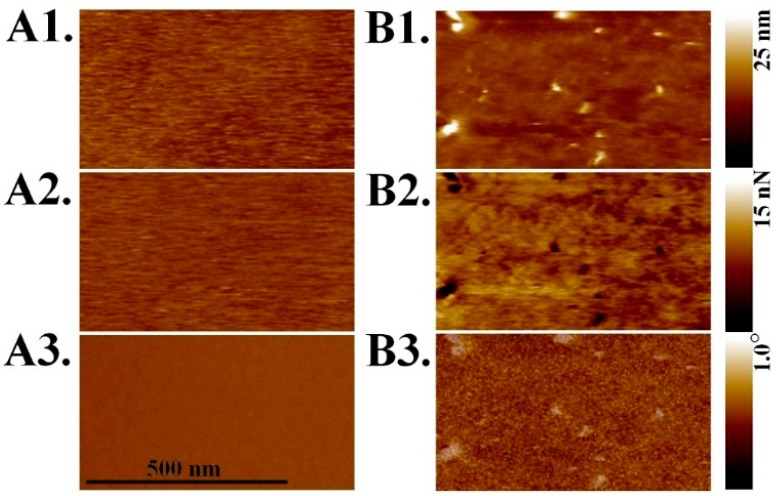
PF-MFM images of poly(APTAC) brushes (**A1**–**A3**) and poly(APTAC)+SPIONs brushes (**B1**–**B3**): 1. topography, 2. adhesion, 3. magnetic phase.

Finally, the magnetic properties of the poly(APTAC)+SPIONs hybrid brushes obtained were studied. Magnetic force microscopy (MFM) confirmed the presence of magnetic domains that can be assigned to the well-separated SPIONs placed in the polymeric matrix (Figure 7). Heterogeneity of the poly(APTAC)+SPIONs sample is also clearly visible in the adhesion image (Figure 7B2) that can be also related to the presence of variously entangled polymer chains at various distances from the embedded SPIONs. Both magnetic signal and adhesion heterogeneity are not visible in the poly(APTAC) sample (Figure 7A2,A3).

## 4. Conclusions

We reported here on a facile and efficient method for homogenous incorporation of magnetic nanoparticles (SPIONs) in the ultrathin (<15 nm) cationic polymer brush layer poly(APTAC) during their formation via surface-initiated polymerization. We showed magnetic signals of the well-separated SPIONs in the formed nanocomposite layer using magnetic force microscopy. A twice higher elastic modulus of the formed SPIONs-containing brushes as compared to the parent poly(APTAC) brushes was indicated using quantitative nanomechanical mapping (QNM) in spite of the low content of SPIONs (ca. 1%). We showed that application of a pulsed sonication during ATRP does not block formation of the surface-grafted brushes while it limits the unwanted aggregation of the added nanoparticles. The proposed method utilizes the nanoparticles formed ex situ, the properties of which can be determined and optimized prior to incorporation of them into a polymer layer. The proposed method can be treated as a model for formation of other hybrid structures composed of charged nanoparticles interacting electrostatically with oppositely charged polymers grafted from surfaces. As such, the method can be useful in the preparation of nanocomposite layers with non-aggregated small nanoparticles well-distributed in a polymeric matrix that are of high interest in e.g., nanoelectronic, photopholtaic applications. 

## Supplementary Materials

The following are available online at https://www.mdpi.com/2079-4991/9/3/456/s1: Figure S1: The exemplary high-resolution transmission electron microscope (HR TEM) image of SPIONs; Figure S2: Histograms of zeta potential values (A.) and hydrodynamic diameter (B.) of the suspension of SPIONs; Figure S3: AFM images of bare SPIONs: A. topography, B.-D. magnetic phase with a lift distance of 50 nm (B.), 100 nm (C.) and 250 nm (D.); Figure S4: The ultraviolet-visible (UV-Vis) absorption spectrum of phenanthroline complex with Fe(II) used for the determination of the iron content in the SPIONs. Inset: the calibration line; Figure S5: AFM images with cross-sections of bare poly(APTAC) brushes (A.) and the same brushes after 5 min of ultrasound treatment in SPIONs suspension (B.); Figure S6: AFM topography images of poly(APTAC)+SPIONs samples after synthesis (A.) and one month (B.); Figure S7: XPS spectrum of poly(APTAC).
