# Supplementary material for: Homogeneous Embedding of Magnetic Nanoparticles into Polymer Brushes during Simultaneous Surface-Initiated Polymerization
Source: Nanomaterials (Basel). 2019 Mar 19;9(3):456. doi: 10.3390/nano9030456 (PMC6474101; doi:10.3390/nano9030456)
Supplement: Supplementary file 1 [file nanomaterials-09-00456-s001.pdf]

Supplementary Materials

Homogeneous embedding of magnetic nanoparticles into polymer brushes during simultaneous surface-initiated polymerization

Weronika Górka [1,2], Tomasz Kuciel [2], Paula Nalepa [2], Dorota Lachowicz [3], Szczepan Zapotoczny [2,*] and Michał Szuwarzyński [3,*]

[1] Faculty of Physics, Astronomy and Applied Computer Science, Jagiellonian University, prof. Stanisława Łojasiewicza 11, 30-348 Krakow, Poland; weronika.gorka@doctoral.uj.edu.pl (W.G.)
[2] Faculty of Chemistry, Jagiellonian University, Gronostajowa 2, 30-387 Krakow, Poland; kuciel@chemia.uj.edu.pl (T.K), paula.nalepa@student.uj.edu.pl (P.N.)
[3] AGH University of Science and Technology, Academic Centre for Materials and Nanotechnology, al. A. Mickiewicza 30, 30-059 Krakow, Poland; dbielska@agh.edu.pl (D.L.)
* Correspondence: szuwarzy@agh.edu.pl (M.S.), zapotocz@chemia.uj.edu.pl (S.Z.);

1. SPIONs characterization

1.1. TEM

TEM imaging was used to determine the size of the obtained SPIONs. The images showed well-separated nanoparticles within small aggregates (Figure S1). The core sizes of the nanoparticles were found to be very small, with an average diameter equal to 8-10 nm.

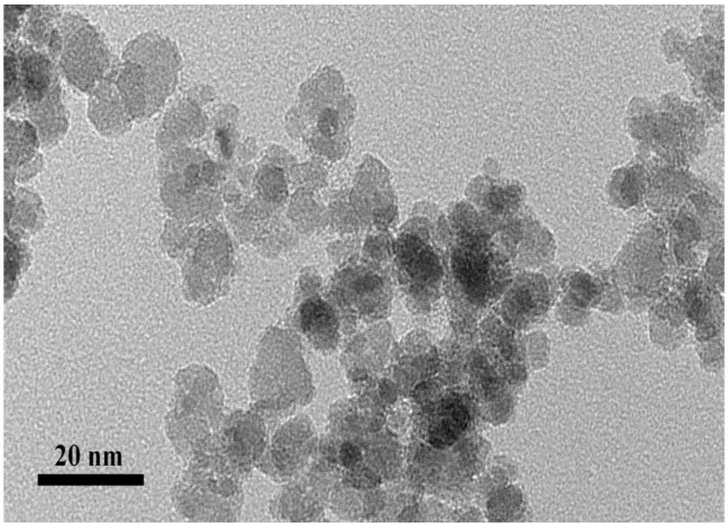

Figure S1. The exemplary HR TEM image of SPIONs.

1.2. Zeta potential and dynamic light scattering measurements

A histogram of zeta potential values measured for SPIONs in the aqueous solution of pH = 9.06 is shown in Figure S2.A. As it can be seen the surface of the nanoparticles is strongly negatively charged (average zeta potential equal to -47.6 ± 0.4 mV). Thus, the suspension can be considered as stable (electrostatic stabilization) because majority of the nanoparticles exhibit highly negative zeta potential values (below -30 mV). Measured hydrodynamic diameter (number distribution, Figure 2.B.) of SPIONs is calculated on 47.69 ± 0.29 nm with PDI equal to 0.162.

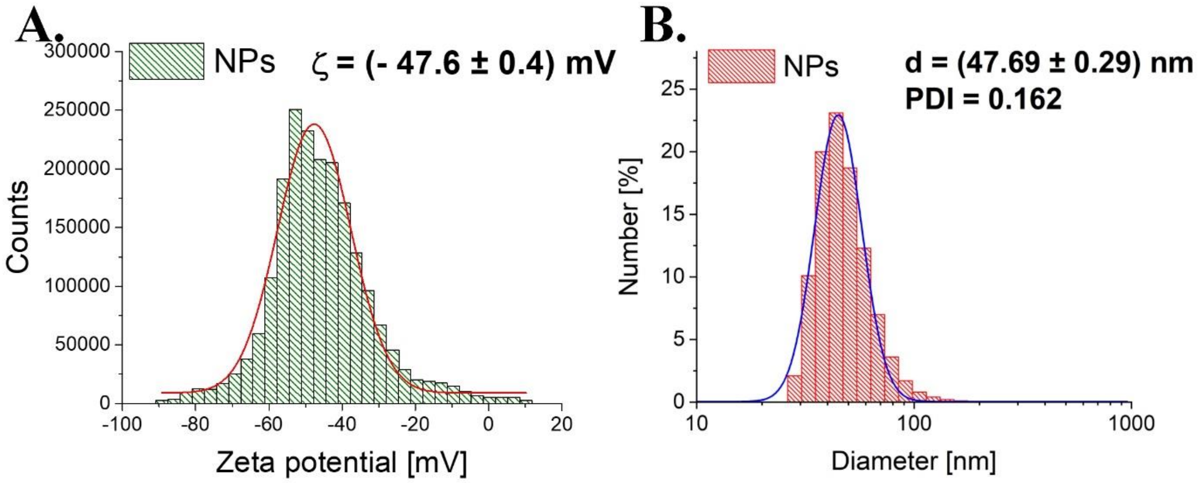

Figure S2. Histograms of zeta potential values (A.) and hydrodynamic diameter (B.) of the suspension of SPIONs.

1.3. Magnetic Force Microscopy (MFM)

Magnetic Force Microscopy was used to confirm the magnetic properties of SPIONs. The images of surface topography and magnetic phase for various lift distance but the same location are presented in Figure 3. They indicate a relatively strong magnetic signal that was still measurable even for the lift distance as large as 250 nm (Figure S3. C) .

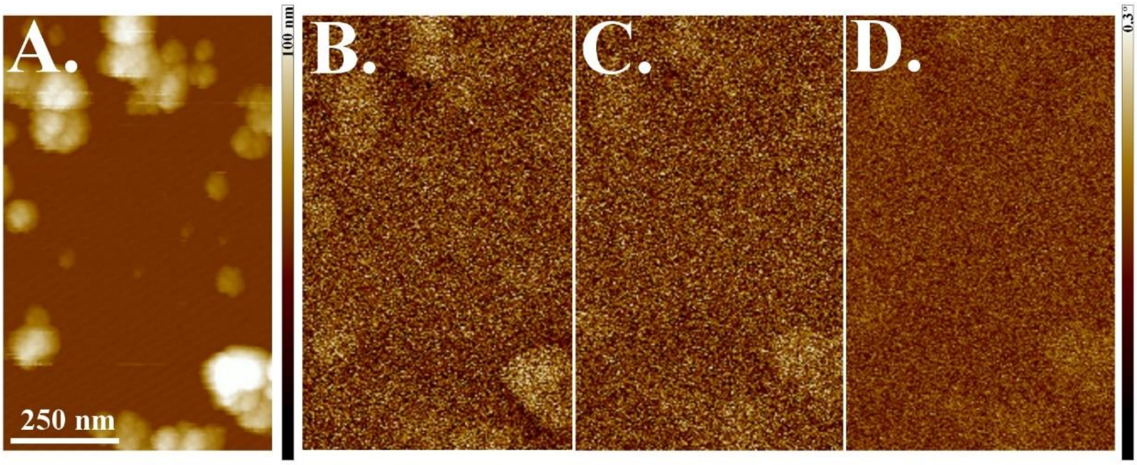

Figure S3. AFM images of bare SPIONs: A. topography , B.-D. magnetic phase with a lift distance of 50 nm (B.), 100 nm (C.) and 250 nm (D.)

1.4. Determination of iron content in the SPION dispersion

Iron content in the nanoparticles dispersion was determined using a classical spectrophotometric method based on absorbance measurements of the color complex of iron(II) with phenanthroline. For this purpose, 3 mL of the nanoparticles dispersion was dissolved in 0.6 mL of 3M HCl. After 24 h, an excess of vitamin C (with respect to the anticipated iron content) was introduced for reducing Fe(III) to Fe(II) and left overnight. Finally, 3 mL of 0.2% solution of phenanthroline in 0.08M HCl was added to the obtained transparent solution. The sample was left for 10 min and its absorbance at the characteristic wavelength, λ_{max} = 512 nm, was measured. Based on the previously prepared calibration, the concentration of iron in the sample was determined to be equal to 0.87 mg/ml (Figure S4).

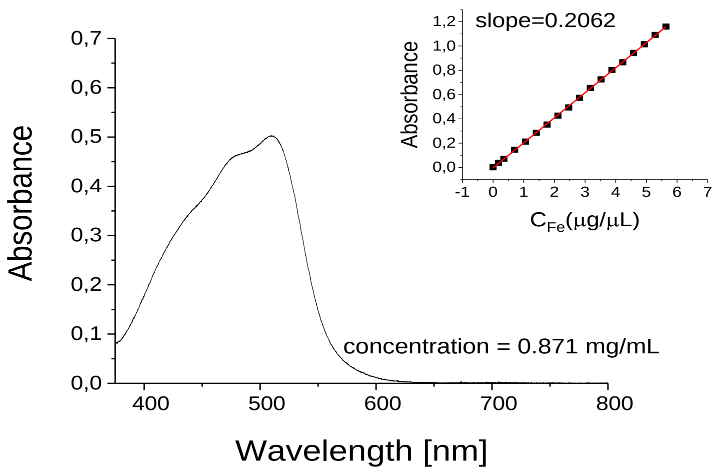

Figure S4. The UV-Vis absorption spectrum of phenanthroline complex with Fe(II) used for the determination of the iron content in the SPIONs. Inset: the calibration line.

2. Deposition of SPIONs onto already prepared poly(APTAC) brushes

A poly(APTAC) brush sample was immersed into the aqueous dispersion of SPIONs and subjected to sonication for 5 min (pulsed sonication as described in 1.3.) in order to check if they can be incorporated into the brushes at the conditions used for the synthesis of poly(APTAC)+SPION. The sample was then withdrawn and rinsed with water prior AFM imaging. The AFM images (Figure S5) showed no differences in brushes topography and thickness before and after the experiment indicating that SPIONs were not incorporated into the brushes at the studied conditions.

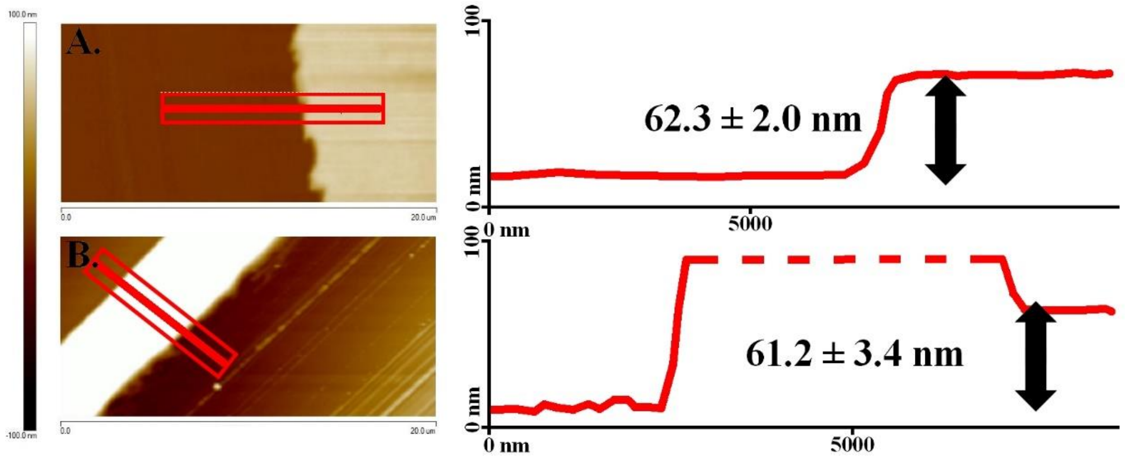

Figure S5. AFM images with cross-sections of bare poly(APTAC) brushes (A.) and the same brushes after 5 min of ultrasound treatment in SPIONs suspension (B.).

3. Stability of poly(APTAC)+SPIONs samples

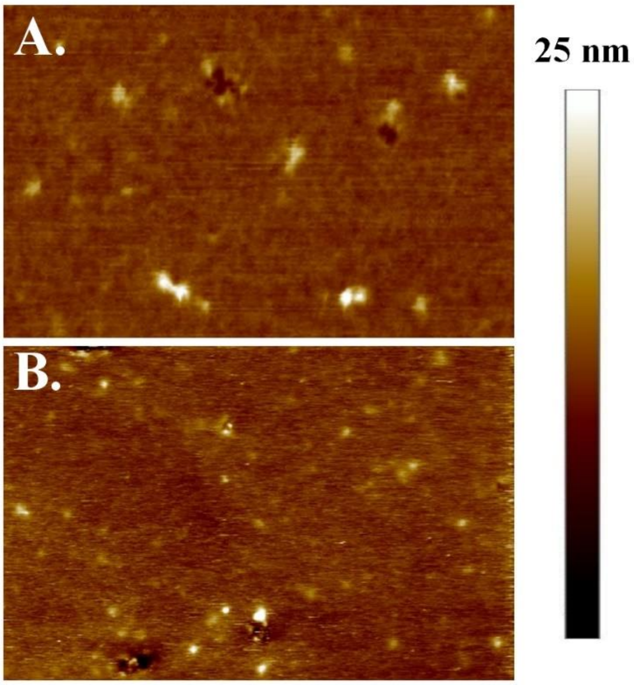

Figure S6. AFM topography images of poly(APTAC)+SPIONs samples after synthesis (A.) and one month (B.).

4. XPS spectrum of poly(APTAC) brushes

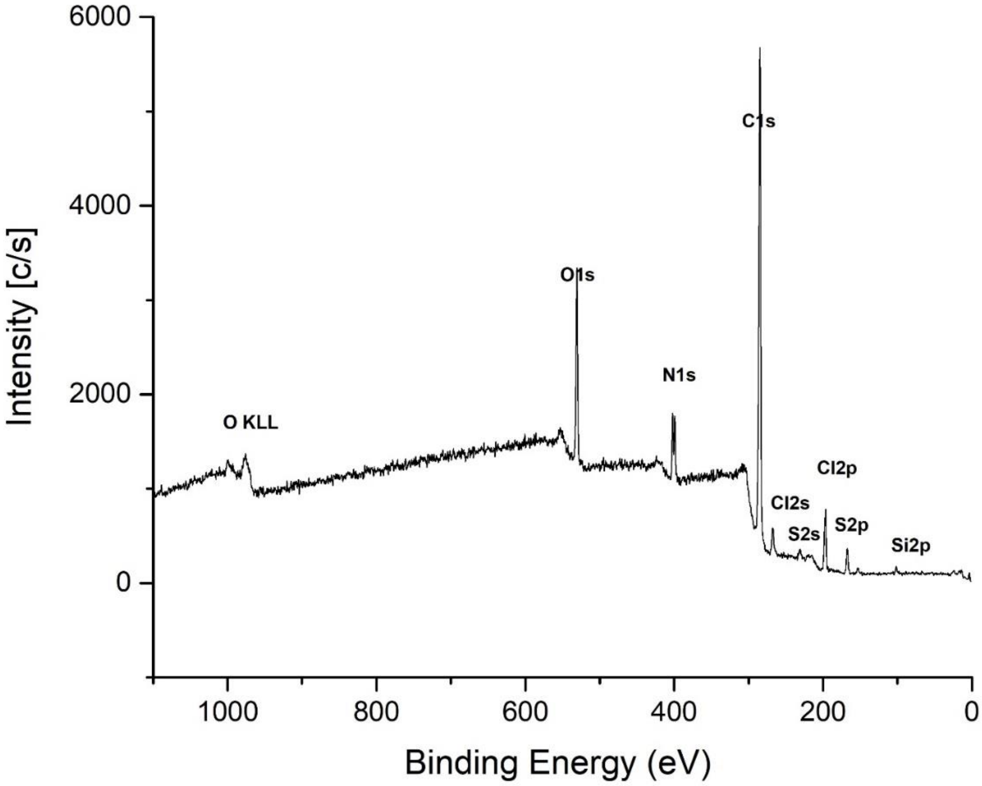

Figure S7. XPS spectrum of poly(APTAC).
